# Association between oxidative balance score and osteosarcopenia in US adults: insights from a population-based study

**DOI:** 10.3389/fnut.2025.1612406

**Published:** 2025-07-31

**Authors:** Zhonghua Sun, Jiahui Yang, Xinyu Tao, Yangyang Weng, Ling Ding, Yameng Xu, Chen Qu, Zhengxia Liu

**Affiliations:** ^1^Department of Geriatrics, The Second Affiliated Hospital of Nanjing Medical University, Nanjing, China; ^2^Department of Pediatrics, The Fourth Affiliated Hospital of Nanjing Medical University, Nanjing, China; ^3^Department of Epidemiology, School of Public Health, Nanjing Medical University, Nanjing, China

**Keywords:** osteosarcopenia, sarcopenia, osteoporosis, antioxidant, oxidative balance score, NHANES

## Abstract

**Background:**

Sarcopenia and osteoporosis are interrelated conditions that significantly contribute to adverse health outcomes. Both conditions are negatively influenced by oxidative stress. While antioxidant supplementation has been explored as a potential intervention, its efficacy remains inconsistent, highlighting the complexity of oxidative stress management. The oxidative balance score (OBS) is a novel metric that evaluates the interplay between pro-oxidants and antioxidants, providing a holistic assessment of the body’s oxidative status. Despite its promise, the relationship between OBS and osteosarcopenia—a coexistence of sarcopenia and osteoporosis—has not been extensively studied.

**Methods:**

Data from the National Health and Nutrition Examination Survey, a national cross-sectional study. The association between OBS and osteosarcopenia was evaluated using weighted logistic regression, restricted cubic spline, and receiver operating characteristic curve analyses. Subgroup analyses were conducted to explore potential population differences. Additionally, we examined the relationships between dietary and lifestyle OBS and osteosarcopenia separately.

**Results:**

The study included 3,336 adults (mean age: 43.30 ± 13.73 years; 53.15% males) with complete data on muscle mass and bone mineral density. OBS was linearly and negatively associated with osteosarcopenia and effectively differentiated individuals with osteosarcopenia from healthy individuals. No significant interactions were observed in the subgroup analyses. Lifestyle OBS showed a stronger association with osteosarcopenia compared to dietary OBS.

**Conclusion:**

OBS is inversely associated with the prevalence of osteosarcopenia, indicating that individuals with higher OBS tend to have a lower likelihood of osteosarcopenia. Moreover, OBS demonstrates good discriminative ability, effectively identifying individuals who are more likely to have osteosarcopenia from healthy individuals.

## Introduction

Osteosarcopenia, characterized by the simultaneous presence of osteoporosis and sarcopenia, manifests in markedly reduced bone mineral density (BMD) and muscle mass. This synergy not only increases bone fragility but also weakens muscle strength, thereby elevating the risk of fractures and falls (1). These conditions together significantly heighten the likelihood of adverse clinical outcomes, including prolonged hospital stays, increased healthcare costs, and reduced quality of life, compared to each condition individually (2). Early detection and preventive strategies are essential for maintaining skeletal and muscular health and are integral to health promotion efforts.

Oxidative stress arises when the generation of reactive oxygen species (ROS) surpasses antioxidant defenses, causing cellular damage and playing a role in the degeneration of muscle and bone tissues (3). Elevated levels of oxidative stress are closely linked to muscle atrophy and bone resorption, crucial factors in the development of sarcopenia and osteoporosis, respectively (4). Moreover, oxidative stress activates signaling pathways that exacerbate the decline in musculoskeletal function (5).

The oxidative balance score (OBS) is a composite measure assessing the balance between pro-oxidants and antioxidants, influenced by dietary and lifestyle factors, reflecting the total oxidative stress burden within the body (6, 7). The OBS has been proven to effectively reflect the level of oxidative stress in diet and lifestyle and has shown robust performance with a variety of diseases, including ischemic heart disease (8, 9), chronic obstructive pulmonary disease (10), non-alcoholic fatty liver disease (11, 12), type 2 diabetes mellitus (13, 14) and oncological diseases (15, 16). Higher OBS values indicate a more favorable antioxidant status and lower levels of oxidative stress (17). Previous studies have confirmed that higher OBS values are associated with reduced prevalence of sarcopenia in adults under 60 years of age and osteoporosis in postmenopausal women (18–21). However, the link between OBS and sarcopenia or osteoporosis across broader age ranges and diverse populations remains underexplored. Moreover, the potential connection between OBS and osteosarcopenia is still unclear.

The National Health and Nutrition Examination Survey (NHANES) provides comprehensive dietary and lifestyle data. Our objective is to use the NHANES dataset to examine the connection between OBS and osteosarcopenia in the adult population of the United States.

## Materials and methods

### Study design and population

This research utilized data obtained from the NHANES, which is publicly accessible on their official website.^1^ The Centers for Disease Control and Prevention (CDC) conducts the NHANES survey biennially, employing a complex, stratified multistage sampling method to ensure the representativeness of the sample.

**FIGURE 1:**
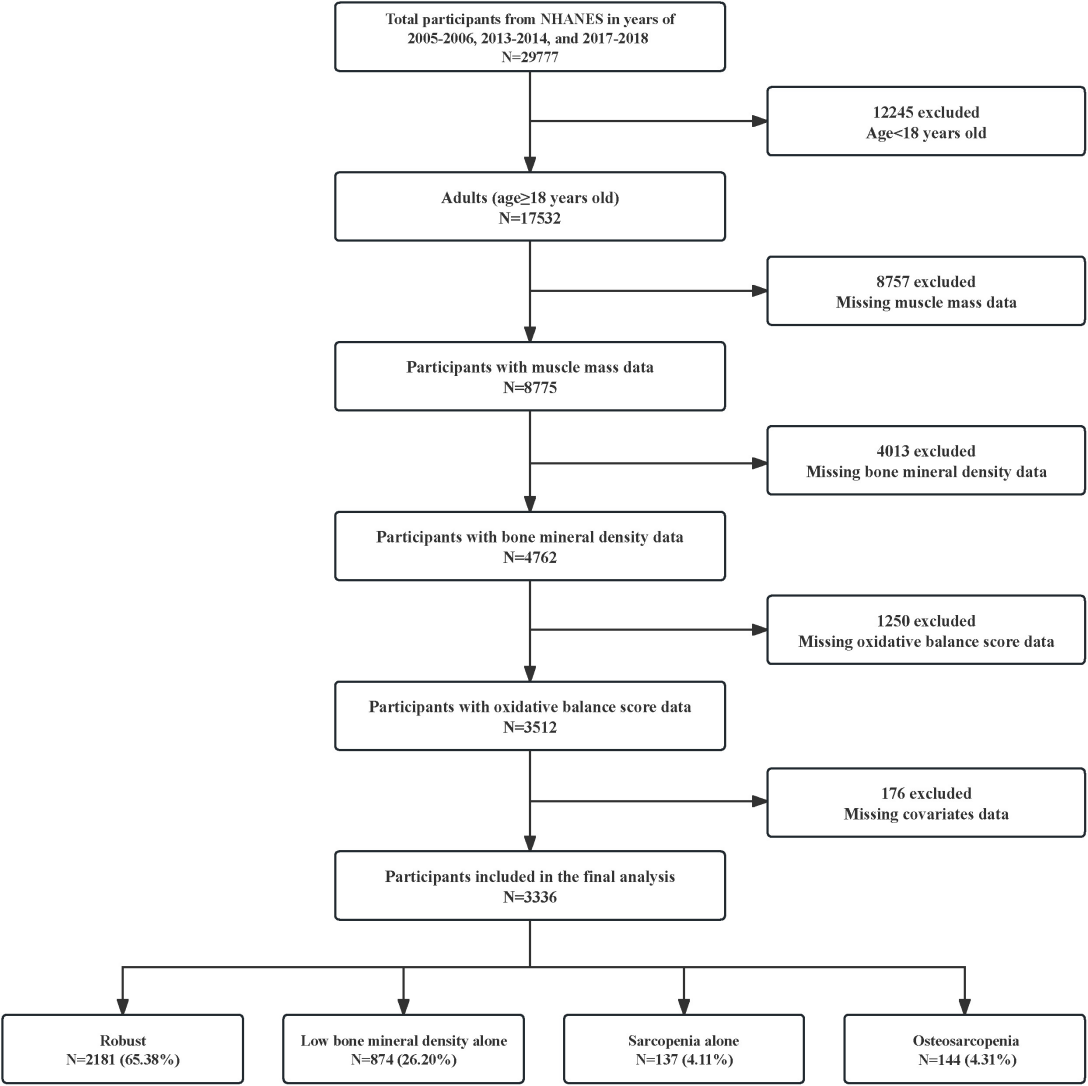
Flowchart for the selection of research participants. NHANES, National Health and Nutrition Examination Survey.

In this study, we reviewed all NHANES data from 1999 to 2020, ultimately selecting three cycles with concurrent muscle mass and BMD measurements (2005–2006, 2013–2014, and 2017–2018). The selection process of study participants is depicted in Figure 1. Out of 29,777 NHANES participants, individuals were excluded for the following reasons: (1) age under 18 years (*N* = 12,245); (2) missing muscle mass data (*N* = 8,757); (3) missing BMD data (*N* = 4,013); (4) missing OBS data (*N* = 1,250); (5) missing covariates data (*N* = 176). Among the 3,336 participants included, we further classified them into four groups: robust (no low BMD and no sarcopenia), low BMD alone (low BMD and no sarcopenia), sarcopenia alone (sarcopenia and no low BMD), and osteosarcopenia (both low BMD and sarcopenia).

### Definition of OBS

The OBS methodology is consistent with those outlined in previous studies (22, 23). In essence, the OBS incorporates 16 dietary components and 4 lifestyle factors. Dietary evaluations are based on data from the 24-h dietary recall interview. Key lifestyle factors included in this scoring system are physical activity, body mass index, levels of alcohol intake, and cotinine concentrations (Supplementary Table 1).

Within the OBS system, components are classified as either antioxidants or prooxidants—with 15 classified as antioxidants and 5 as prooxidants. As shown in Supplementary Table 1, the 20 components are stratified by sex (male/female), and each component is divided into tertiles based on sex-specific distributions. For antioxidant components, scores of 0, 1, and 2 are assigned from the lowest to highest tertile, while for prooxidant components, the scoring is reversed (2, 1, 0 from lowest to highest tertile). Alcohol consumption is scored based on established thresholds in the literature: non-drinkers (score = 2), non-heavy drinkers (≤ 15 g/day for women, ≤ 30 g/day for men; score = 1), and heavy drinkers (> 15 g/day for women, > 30 g/day for men; score = 0).

### Diagnosis of the osteosarcopenia phenotype

BMD and appendicular skeletal muscle mass (ASM) are assessed using dual-energy X-ray absorptiometry (DXA). A BMD value less than one standard deviation below the reference value is considered low BMD (osteopenia or osteoporosis). Sarcopenia is defined using the appendicular skeletal muscle mass index (ASMI), calculated as ASMI = ASM/body mass index (BMI), with diagnostic cutoff values of < 0.512 for females and < 0.789 for males. Osteosarcopenia is defined as the concurrent presence of both low BMD and sarcopenia (24–26).

### Covariate assessment

Considering potential confounders that might affect the OBS and osteosarcopenia, we identified the following covariates based on clinical expertise and existing literature (27, 28): age (categorized as 18–44 years, 45–59 years, and ≥ 60 years), gender (male and female), race (non-Hispanic White and other), marital status (married/living with a partner and other), Poverty Income Ratio (PIR) (grouped into < 1.3, 1.3–3.5, and ≥ 3.5), education level (divided into < High school, High school, and > High school), and medical conditions (including hypertension, diabetes, and hyperlipidemia). Hypertension was identified by a self-reported physician’s diagnosis, systolic blood pressure of 140 mmHg or higher, diastolic blood pressure of 90 mmHg or more, or the use of antihypertensive medication (29). Diabetes was diagnosed based on a self-reported physician’s diagnosis, a glycated hemoglobin A1c level of 6.5% or greater, the use of insulin or other diabetes medications, a fasting glucose level of 7.0 mmol/L or higher, a random glucose level above 11.1 mmol/L, or an oral glucose tolerance test result of 11.1 mmol/L or more (30). Hyperlipidemia was confirmed if total cholesterol was 200 mg/dL or higher, triglycerides were above 150 mg/dL, low-density lipoprotein cholesterol was 130 mg/dL or greater, or high-density lipoprotein cholesterol was below 40 mg/dL (31).

### Statistical analyses

In the analysis of continuous variables, data are presented as the mean ± standard deviation (SD). The one-way analysis of variance is used to assess differences between groups. For categorical variables, data are presented as frequencies (*n*) and percentages (%), with disparities among groups evaluated using the chi-square test.

To enhance the robustness of our analysis, we employed a weighting strategy (i.e., 1/3 * WTDRD1) in exploring the relationship between the OBS and osteosarcopenia phenotypes (32). We used several weighted logistic regression models to assess the association between OBS and osteosarcopenia phenotypes, categorizing OBS into quartiles to enhance robustness. Model 1: unadjusted; Model 2: adjusted for demographic and socioeconomic covariates (age group, gender, race, marital status, poverty-income ratio, and education level); Model 3: additionally adjusted for hypertension, diabetes, and hyperlipidemia. Subsequently, we utilized Restricted cubic spline (RCS) analysis with three knots points to investigate the dose-response relationship between OBS and osteosarcopenia phenotypes. To further assess the predictive accuracy of OBS for osteosarcopenia phenotypes, a nomogram model and a receiver operating characteristic (ROC) curve were constructed using OBS combined with demographic covariates. Subgroup analysis was conducted to explore potential heterogeneity in the association between OBS and osteosarcopenia phenotypes across different subgroups. To identify the key components of OBS that affect osteosarcopenia, we separately examined the associations of dietary OBS and lifestyle OBS with osteosarcopenia phenotypes. Finally, we used weighted linear regression to explore the relationship between different osteosarcopenia phenotypes and OBS levels.

All analyses were conducted using R software, version 4.1.0 (R Foundation for Statistical Computing). A two-sided *P*-value below 0.05 was considered statistically significant.

## Results

### Population characteristics

**FIGURE 2:**
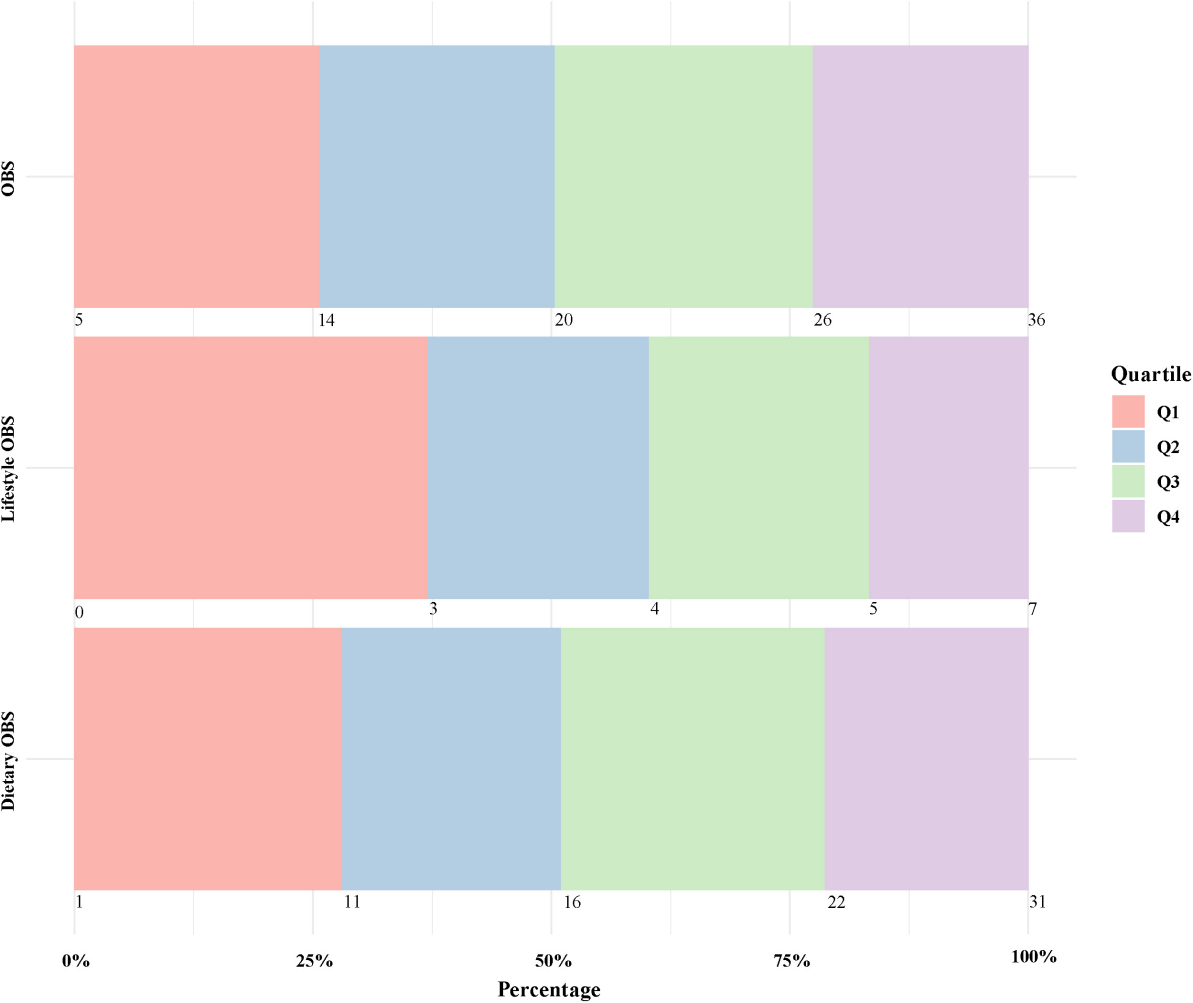
Specific ranges and proportions of oxidative balance score quartiles. OBS, oxidative balance score quartiles.

A total of 3,336 participants with complete data were included in the study. The average age of the participants was 43.3 years, and males constituted 53.15% of the cohort. Based on the osteosarcopenia phenotype, participants were divided into groups as shown in Table 1. The mean ages for the robust, low BMD alone, sarcopenia alone, and osteosarcopenia groups were 41.43 ± 13.90, 45.70 ± 12.64, 50.31 ± 12.78, and 50.56 ± 11.44 years, respectively. Compared to the robust group, the osteosarcopenia group had fewer non-Hispanic White, lower socioeconomic and education levels, and higher prevalence of hypertension, diabetes, and hyperlipidemia. Figure 2 displayed the specific ranges and proportions of OBS quartiles, similar to other OBS studies that were published using NHANES data (32, 33).

**TABLE 1:** Initial characteristics of the study participants.

Variables	Total	Robust	Low BMD	Sarcopenia	Osteosarcopenia	*P*-value
	(*N* = 3,336)	(*N* = 2,181)	(*N* = 874)	(*N* = 137)	(*N* = 144)	
**Age (year)**	43.30 ± 13.73	41.43 ± 13.90	45.70 ± 12.64	50.31 ± 12.78	50.56 ± 11.44	**< 0.001**
**Age group**						**< 0.001**
18–44	1,568 (47.00%)	1,161 (53.23%)	331 (37.87%)	38 (27.74%)	38 (26.39%)	
45–59	1,465 (43.91%)	858 (39.34%)	468 (53.55%)	64 (46.72%)	75 (52.08%)	
≥ 60	303 (9.08%)	162 (7.43%)	75 (8.58%)	35 (25.55%)	31 (21.53%)	
**Gender**						0.330
Female	1,563 (46.85%)	1,028 (47.13%)	407 (46.57%)	55 (40.15%)	73 (50.69%)	
Male	1,773 (53.15%)	1,153 (52.87%)	467 (53.43%)	82 (59.85%)	71 (49.31%)	
**Race**						**< 0.001**
Non-Hispanic White	1,506 (45.14%)	1,014 (46.49%)	409 (46.80%)	47 (34.31%)	36 (25.00%)	
Other	1,830 (54.86%)	1,167 (53.51%)	465 (53.20%)	90 (65.69%)	108 (75.00%)	
**Marital status**						0.053
Married/living with a partner	2,063 (61.84%)	1,315 (60.29%)	558 (63.84%)	92 (67.15%)	98 (68.06%)	
Other	1,273 (38.16%)	866 (39.71%)	316 (36.16%)	45 (32.85%)	46 (31.94%)	
**PIR**						**< 0.001**
< 1.3	836 (25.06%)	504 (23.11%)	231 (26.43%)	43 (31.39%)	58 (40.28%)	
1.3–3.5	1,161 (34.80%)	749 (34.34%)	308 (35.24%)	55 (40.15%)	49 (34.03%)	
≥ 3.5	1,339 (40.14%)	928 (42.55%)	335 (38.33%)	39 (28.47%)	37 (25.69%)	
**Education level**						**< 0.001**
< High school	633 (18.97%)	347 (15.91%)	191 (21.85%)	44 (32.12%)	51 (35.42%)	
High school	812 (24.34%)	530 (24.30%)	211 (24.14%)	38 (27.74%)	33 (22.92%)	
> High school	1,891 (56.68%)	1,304 (59.79%)	472 (54.00%)	55 (40.15%)	60 (41.67%)	
**Hypertension**						**< 0.001**
No	2,356 (70.62%)	1,563 (71.66%)	621 (71.05%)	74 (54.01%)	98 (68.06%)	
Yes	980 (29.38%)	618 (28.34%)	253 (28.95%)	63 (45.99%)	46 (31.94%)	
**Diabetes**						**< 0.001**
No	2,953 (88.52%)	1,967 (90.19%)	784 (89.70%)	91 (66.42%)	111 (77.08%)	
Yes	383 (11.48%)	214 (9.81%)	90 (10.30%)	46 (33.58%)	33 (22.92%)	
**Hyperlipidemia**						**< 0.001**
No	1,130 (33.87%)	826 (37.87%)	253 (28.95%)	26 (18.98%)	25 (17.36%)	
Yes	2,206 (66.13%)	1,355 (62.13%)	621 (71.05%)	111 (81.02%)	119 (82.64%)	
**OBS**	20.13 ± 7.06	20.31 ± 7.06	20.35 ± 7.01	17.77 ± 6.99	18.35 ± 6.87	**< 0.001**
**Dietary OBS**	16.12 ± 6.75	16.31 ± 6.77	16.14 ± 6.70	14.42 ± 6.57	14.65 ± 6.46	**0.001**
**Lifestyle OBS**	4.02 ± 1.51	4.01 ± 1.52	4.20 ± 1.49	3.36 ± 1.51	3.71 ± 1.36	**< 0.001**

Age and OBS are continuous variables presented as mean ± standard deviation (SD). All other variables are categorical, presented as frequencies (percentages). Bold indicates a *P*-value < 0.05. OBS, oxidative balance score; PIR, poverty income ratio; BMD, bone mineral density.

### Association between OBS and osteosarcopenia phenotypes

Weighted logistic regression analysis revealed potential associations between OBS and both sarcopenia and osteosarcopenia [in Model 3, the odds ratios (ORs) with 95% confidence intervals (CI) were 0.94 (0.91, 0.97) and 0.96 (0.92, 1.00), respectively]. This negative correlation remained stable across other adjusted and quantile models. We observed no significant link between OBS and low BMD in our research (Table 2).

**TABLE 2:** Weighted logistic regression analysis on the associations between OBS and osteosarcopenia phenotype.

Variables	Low BMD		Sarcopenia		Osteosarcopenia	
	OR (95% CI)	*P*-value	OR (95% CI)	*P*-value	OR (95% CI)	*P*-value
**Model 1**						
**OBS**	1.00 (0.98, 1.01)	0.564	0.92 (0.90, 0.95)	**< 0.001**	0.95 (0.92, 0.98)	**0.002**
Q1	Ref		Ref		Ref	
Q2	1.15 (0.83, 1.59)	0.408	0.63 (0.31, 1.30)	0.208	0.82 (0.44, 1.51)	0.510
Q3	1.03 (0.75, 1.41)	0.873	0.60 (0.34, 1.06)	0.079	0.74 (0.42, 1.30)	0.289
Q4	0.95 (0.70, 1.29)	0.738	0.15 (0.08, 0.30)	**< 0.001**	0.21 (0.10, 0.46)	**< 0.001**
*P* for trend		0.608		**< 0.001**		**< 0.001**
**Model 2**						
**OBS**	1.00 (0.99, 1.02)	0.982	0.94 (0.91, 0.97)	**< 0.001**	0.94 (0.91, 0.97)	**< 0.001**
Q1	Ref		Ref		Ref	
Q2	1.27 (0.90, 1.78)	0.167	0.73 (0.35, 1.50)	0.381	0.73 (0.35, 1.50)	0.381
Q3	1.08 (0.77, 1.52)	0.628	0.78 (0.42, 1.46)	0.429	0.78 (0.42, 1.46)	0.429
Q4	1.04 (0.75, 1.44)	0.799	0.19 (0.10, 0.38)	**< 0.001**	0.19 (0.10, 0.38)	**< 0.001**
*P* for trend		0.955		**< 0.001**		**0.005**
**Model 3**						
**OBS**	1.00 (0.99, 1.02)	0.921	0.94 (0.91, 0.97)	**< 0.001**	0.96 (0.92, 1.00)	**0.033**
Q1	Ref		Ref		Ref	
Q2	1.29 (0.92, 1.81)	0.139	0.70 (0.33, 1.49)	0.343	0.86 (0.46, 1.62)	0.639
Q3	1.10 (0.78, 1.55)	0.574	0.79 (0.43, 1.47)	0.452	0.85 (0.47, 1.54)	0.582
Q4	1.05 (0.76, 1.46)	0.741	0.20 (0.10, 0.39)	**< 0.001**	0.25 (0.11, 0.56)	**0.001**
*P* for trend		0.983		**< 0.001**		**0.006**

Model 1: Unadjusted model. Model 2: Adjusted for age group, gender, race, marital status, poverty-income ratio, and education level. Model 3: Additionally, adjusted for hypertension, diabetes, and hyperlipidemia. Bold indicates a *P*-value < 0.05. OBS, oxidative balance score; OR, odds ratio; CI, confidence interval; BMD, bone mineral density.

### RCS analysis

**FIGURE 3:**
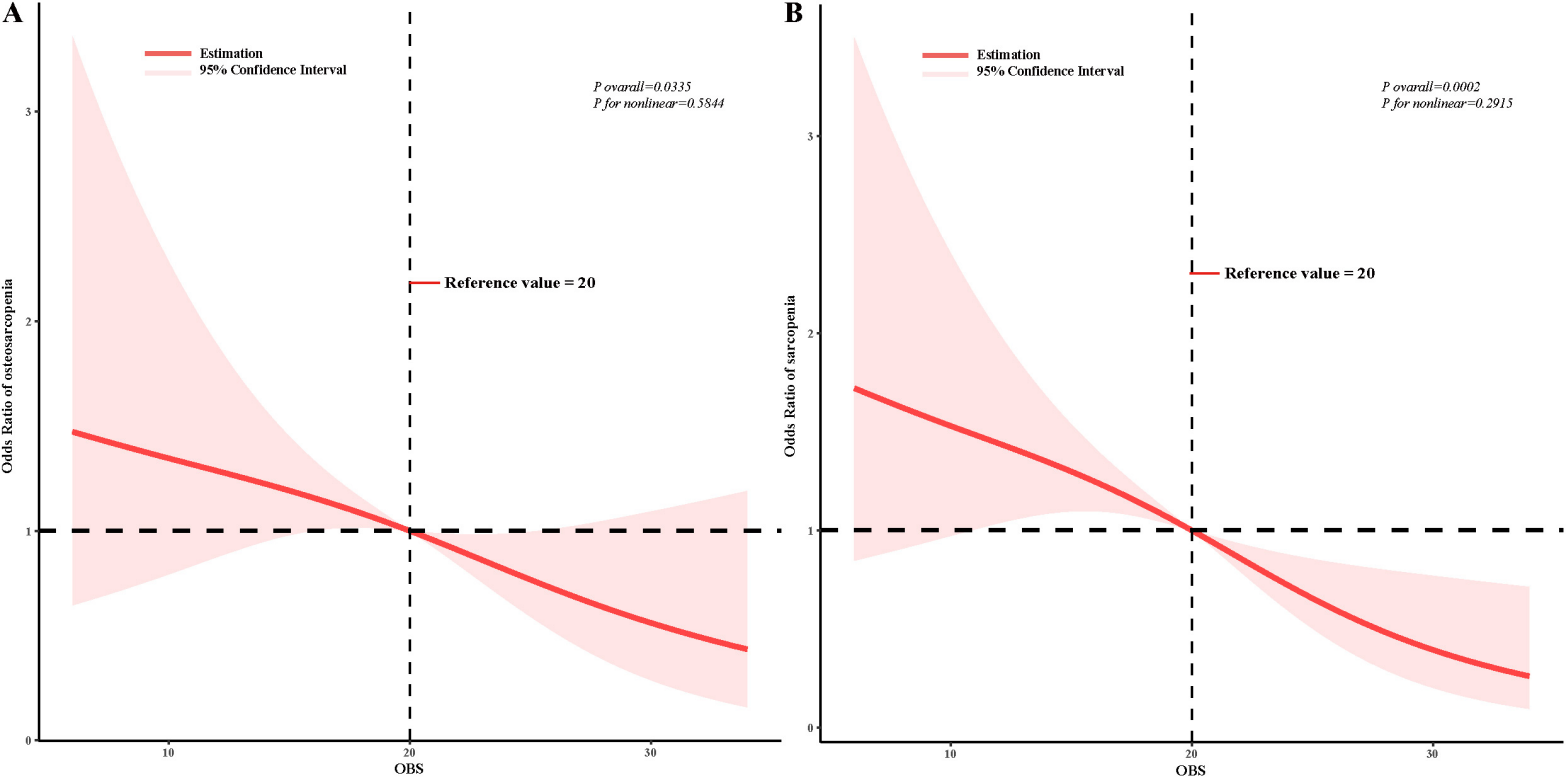
Restricted cubic spline analysis of the oxidative balance score with sarcopenia and osteosarcopenia. The association was adjusted for age group, gender, race, marital status, poverty income ratio, education level, hypertension, diabetes, and hyperlipidemia. Reference values are defined as the median; OBS, oxidative balance score. **(A)** OBS and osteosarcopenia; **(B)** OBS and sarcopenia.

The results of our RCS analysis indicate that the relationships between OBS and both sarcopenia (*P* overall = 0.0002, *P* non-linear = 0.2915) and osteosarcopenia (*P* overall = 0.0335, *P* non-linear = 0.5844) are linear (Figures 3A, B).

### Nomogram model and ROC curve

**FIGURE 4:**
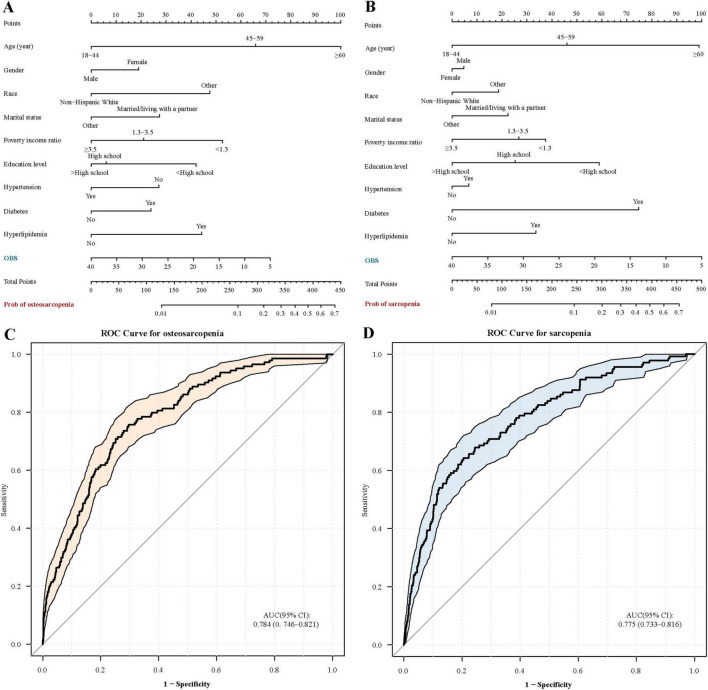
Nomograms and receiver operating characteristic curves for assessing sarcopenia and osteosarcopenia. OBS, oxidative balance score. **(A)** Nomogram for osteosarcopenia; **(B)** Nomogram for sarcopenia; **(C)** ROC curve for osteosarcopenia; **(D)** ROC curve for sarcopenia.

Our nomogram analysis showed that the OBS significantly influences both osteosarcopenia and sarcopenia (Figures 4A, B). The ROC curve analysis further indicated that the area under the curve (AUC) for osteosarcopenia and sarcopenia was 78.4% (95% CI: 74.6%–82.1%) and 77.5% (95% CI: 73.3%–81.6%), respectively (Figures 4C, D). These results indicated that OBS was an effective indicator for distinguishing individuals with osteosarcopenia or sarcopenia from robust individuals.

### Subgroup analysis

**FIGURE 5:**
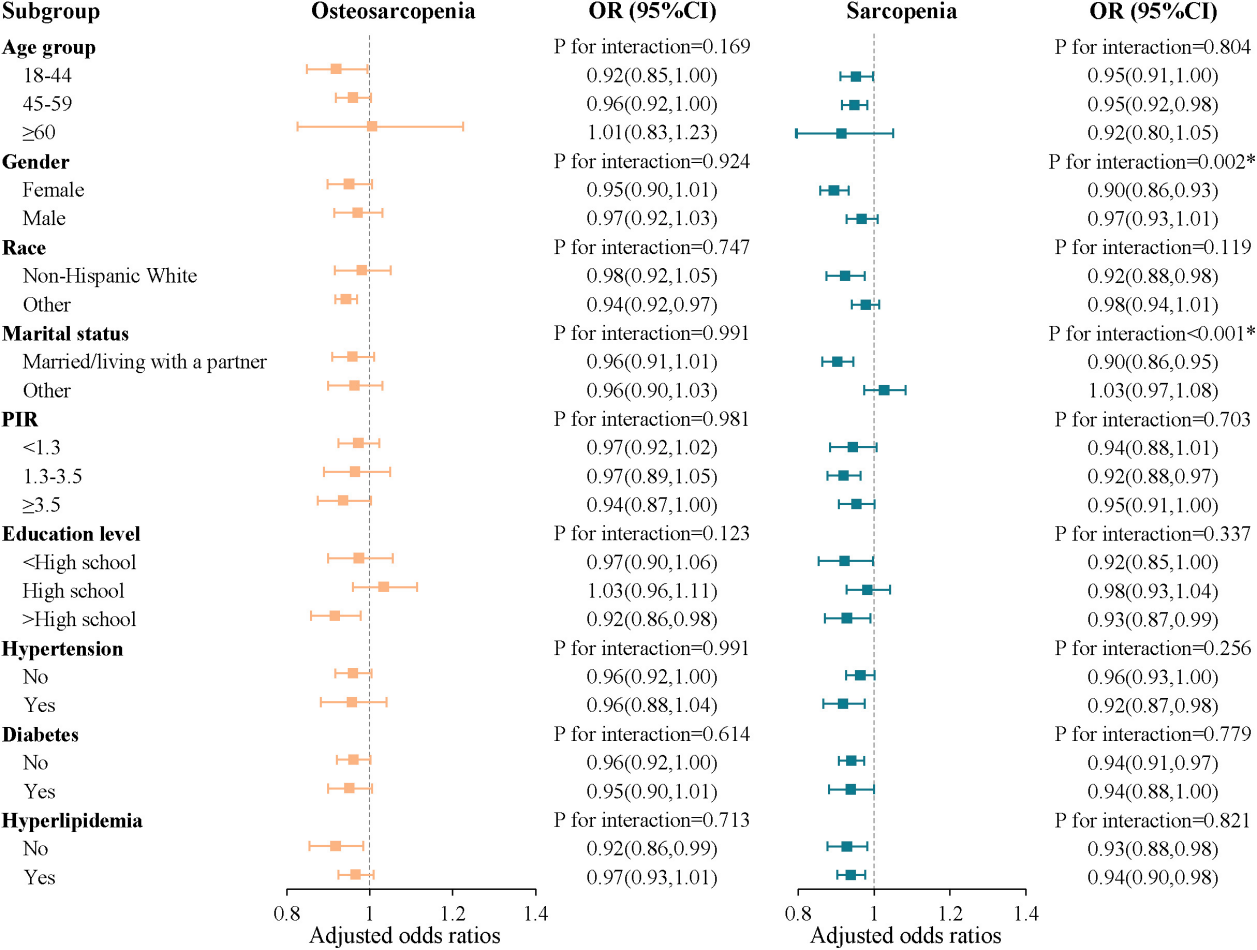
Subgroup analysis of the oxidative balance score with sarcopenia and osteosarcopenia. The symbol “*” denotes *P* < 0.05 for interaction; OR, odds ratio; CI, confidence intervals; PIR, poverty income ratio.

Subgroup analyses indicated a consistent negative correlation between OBS and osteosarcopenia across various subgroups, including age group, gender, race, marital status, poverty income ratio, education level, hypertension, diabetes, and hyperlipidemia (*P* for interaction > 0.05). Additionally, our findings indicated that gender (*P* for interaction = 0.002) and marital status (*P* for interaction < 0.001) might have acted as potential modifiers in the relationship between OBS and sarcopenia (Figure 5).

### Association between dietary OBS/lifestyle OBS and osteosarcopenia phenotypes

Upon categorizing the components of OBS, significant associations were observed between dietary OBS, lifestyle OBS, and sarcopenia across various models [in Model 3, the OR with 95% CI was 0.95 (0.92, 0.98) for dietary OBS and 0.68 (0.56, 0.83) for lifestyle OBS]. Conversely, the link between dietary OBS and osteosarcopenia lacked significance [in Model 3, the OR with 95% CI was 0.96 (0.93, 1.00)]. No significant associations were observed between dietary OBS and lifestyle OBS with low BMD (Table 3).

**TABLE 3:** Weighted logistic regression analysis on the associations between dietary OBS/lifestyle OBS and osteosarcopenia phenotype.

Variables	Low BMD		Sarcopenia		Osteosarcopenia	
	OR (95% CI)	*P*-value	OR (95% CI)	*P*-value	OR (95% CI)	*P*-value
**Model 1**						
**Dietary OBS**	0.99 (0.98, 1.01)	0.351	0.94 (0.91, 0.96)	**< 0.001**	0.95 (0.92, 0.99)	**0.010**
Q1	Ref		Ref		Ref	
Q2	1.10 (0.79, 1.55)	0.564	0.74 (0.36, 1.54)	0.416	0.63 (0.33, 1.20)	0.155
Q3	0.94 (0.67, 1.32)	0.735	0.69 (0.39, 1.23)	0.204	0.72 (0.40, 1.30)	0.273
Q4	1.02 (0.78, 1.32)	0.889	0.24 (0.11, 0.51)	**< 0.001**	0.28 (0.11, 0.69)	**0.007**
*P* for trend		0.774		**0.001**		**0.009**
**Lifestyle OBS**	1.05 (0.99, 1.11)	0.105	0.66 (0.56, 0.79)	**< 0.001**	0.82 (0.71, 0.94)	**0.004**
Q1	Ref		Ref		Ref	
Q2	1.35 (0.90, 2.02)	0.146	0.54 (0.27, 1.09)	0.085	0.73 (0.36, 1.48)	0.371
Q3	1.33 (0.96, 1.83)	0.083	0.38 (0.17, 0.87)	**0.023**	0.68 (0.39, 1.17)	0.162
Q4	1.18 (0.87, 1.59)	0.281	0.11 (0.05, 0.26)	**< 0.001**	0.22 (0.08, 0.59)	**0.003**
*P* for trend		0.116		**< 0.001**		**0.001**
**Model 2**						
**Dietary OBS**	1.00 (0.98, 1.01)	0.742	0.95 (0.92, 0.98)	**0.002**	0.96 (0.93, 1.00)	0.073
Q1	Ref		Ref		Ref	
Q2	1.18 (0.84, 1.67)	0.329	0.84 (0.39, 1.85)	0.662	0.63 (0.31, 1.29)	0.198
Q3	0.97 (0.68, 1.37)	0.855	0.92 (0.49, 1.74)	0.799	0.84 (0.45, 1.57)	0.577
Q4	1.13 (0.85, 1.49)	0.402	0.30 (0.14, 0.62)	**0.002**	0.32 (0.12, 0.85)	**0.023**
*P* for trend		0.803		**0.016**		0.052
**Lifestyle OBS**	1.06 (1.00, 1.12)	0.062	0.67 (0.56, 0.80)	**< 0.001**	0.81 (0.70, 0.94)	**0.005**
Q1	Ref		Ref		Ref	
Q2	1.37 (0.94, 2.00)	0.097	0.54 (0.27, 1.08)	0.078	0.71 (0.33, 1.55)	0.381
Q3	1.29 (0.94, 1.77)	0.114	0.38 (0.16, 0.88)	**0.025**	0.66 (0.36, 1.21)	0.175
Q4	1.22 (0.91, 1.63)	0.179	0.12 (0.05, 0.31)	**< 0.001**	0.21 (0.08, 0.53)	**0.002**
*P* for trend		0.100		**< 0.001**		**0.001**
**Model 3**						
**Dietary OBS**	1.00 (0.98, 1.01)	0.775	0.95 (0.92, 0.98)	**0.003**	0.96 (0.93, 1.00)	0.078
Q1	Ref		Ref		Ref	
Q2	1.18 (0.84, 1.67)	0.335	0.82 (0.37, 1.81)	0.607	0.63 (0.31, 1.28)	0.194
Q3	0.97 (0.68, 1.38)	0.876	0.91 (0.48, 1.74)	0.776	0.83 (0.45, 1.54)	0.551
Q4	1.13 (0.86, 1.50)	0.367	0.30 (0.14, 0.63)	**0.003**	0.33 (0.13, 0.86)	**0.025**
*P* for trend		0.757		**0.022**		0.052
**Lifestyle OBS**	1.06 (1.00, 1.13)	**0.038**	0.68 (0.56, 0.83)	**< 0.001**	0.82 (0.71, 0.96)	**0.014**
Q1	Ref		Ref		Ref	
Q2	1.43 (0.97, 2.09)	0.068	0.51 (0.25, 1.04)	0.062	0.74 (0.35, 1.58)	0.430
Q3	1.32 (0.96, 1.82)	0.081	0.40 (0.17, 0.95)	**0.039**	0.69 (0.37, 1.29)	0.236
Q4	1.24 (0.92, 1.67)	0.146	0.13 (0.05, 0.36)	**< 0.001**	0.21 (0.08, 0.55)	**0.002**
*P* for trend		0.070		**0.002**		**0.003**

Model 1: Unadjusted model. Model 2: Adjusted for age group, gender, race, marital status, poverty-income ratio, and education level. Model 3: Additionally, adjusted for hypertension, diabetes, and hyperlipidemia. Bold indicates a *P*-value < 0.05; OBS, oxidative balance score; OR, odds ratio; CI, confidence interval; BMD, bone mineral density.

### Association between osteosarcopenia phenotypes and OBS levels

Further analyses were conducted to examine OBS level variations across different osteosarcopenia phenotypes (Supplementary Table 2). In the fully adjusted Model 3, participants with osteosarcopenia had OBS levels 1.75 units lower (β: −1.75, 95% CI: −3.43 to −0.06) than those in the robust group, and those with sarcopenia showed a reduction of 2.68 units (β: −2.68, 95% CI: −4.13 to −1.22). No significant association was found between low BMD and OBS levels.

### Sensitivity analysis

To further validate our findings, we conducted additional multivariate logistic regression models based on Model 3. We separately added three key covariates: dietary supplement use (No/Yes), vitamin D status [non-deficient/deficient, determined by total serum 25(OH)D levels via HPLC–MS/MS], and inflammation markers (SIRI, SII, and AISI—novel indices reflecting systemic inflammation and immune activation). These adjustments formed Models 4 through 6. In Model 7, we simultaneously included all three covariates. To ensure the accuracy and consistency of the sensitivity analyses, we excluded five participants with missing values for these additional covariates. The final analytic sample for Models 4–7 therefore consisted of individuals with complete data on all variables included in the respective models. Across all models, OBS remained inversely associated with osteosarcopenia prevalence (Supplementary Table 3).

## Discussion

In this cross-sectional study based on 3,336 NHANES participants (2005–2018), we observed a linear inverse association between OBS values and the prevalence of both osteosarcopenia and sarcopenia. Furthermore, ROC curve analyses suggested that OBS has good discriminatory ability for identifying individuals with these conditions.

Oxidative stress is known to damage muscle cells, impairing their function and quality, and disrupting the balance between osteoblasts and osteoclasts, which adversely affects bone health (34–36). Moderate physical exercise can promote muscle and bone health by reducing oxidative stress levels (37). However, some studies suggest that antioxidants may exhibit pro-oxidant properties under certain conditions (38–40). Therefore, it is essential to comprehensively assess the combined influence of dietary and lifestyle-derived exogenous pro- and antioxidants on the prevalence of osteosarcopenia. Our findings suggest that higher antioxidant exposure, as reflected by diet and lifestyle OBS, is associated with a lower prevalence of osteosarcopenia.

After categorizing the components of the OBS, we found that both dietary OBS and lifestyle OBS were significantly negatively correlated with the prevalence of sarcopenia. However, among individuals with osteosarcopenia, only lifestyle OBS showed a significant association. This result may be attributed to the higher metabolic rate and oxygen demand of muscle cells compared to bone cells, making them more susceptible to oxidative stress (41, 42). Additionally, the cumulative effects of lifestyle improvements may be more pronounced for individuals with osteosarcopenia than those of dietary adjustments. Although this study did not find a significant association between dietary OBS and osteosarcopenia prevalence, moderate intake of certain antioxidants in individuals with osteosarcopenia remains important for several reasons: (1) A comprehensive OBS requires contributions from dietary OBS to achieve optimal levels of antioxidants that benefit muscle and bone health; (2) Intake of certain antioxidants, such as calcium, vitamin E, and B vitamins, has been shown to improve muscle and bone health by reducing oxidative stress levels (43–45); (3) Many dietary antioxidants possess anti-inflammatory properties, which can indirectly mitigate the adverse effects of inflammatory mediators on the body (46–48).

In our study, we identified some differences compared to other related research. The association between the OBS and sarcopenia in our findings aligns with two studies conducted in the US population (18, 19); however, no significant correlation was observed in the Iranian population (49). Potential reasons for this discrepancy include the small sample size in the Iranian study, which may introduce bias, and the older age of participants, leading to better dietary habits and lifestyle choices that result in a reduced impact of OBS changes (28). Finally, the influence of OBS on sarcopenia may vary significantly across different populations. Additionally, we observed a strong association between OBS and osteoporosis in postmenopausal women and adults under 40 (21, 50). This difference may arise from these populations experiencing poorer oxidative stress status, along with the potential presence of sarcopenia among individuals with osteoporosis.

Our subgroup analysis showed that the inverse relationship between OBS and sarcopenia was more prominent in females and in participants who were married or cohabiting with a partner. These patterns may reflect both biological and behavioral mechanisms. In females, particularly those undergoing menopausal transition or postmenopause, estrogen—a key hormone with antioxidant and anti-inflammatory properties—tends to decline, which may increase vulnerability to oxidative stress and sarcopenia. This may amplify the beneficial effects of antioxidant exposures in this population (51, 52). In addition, marital status is closely linked to consistent health-related behaviors. Individuals who are married or partnered are more likely to maintain healthier dietary patterns and exhibit better adherence to health-promoting habits, which may contribute to higher OBS levels and, consequently, a reduced risk of sarcopenia (53).

This study offers several notable advantages. First, it is the first to identify an association between the OBS and the prevalence of osteosarcopenia, providing a comprehensive index for assessing the impact of exogenous antioxidants on this condition. Second, we employed multiple modeling approaches and sensitivity analyses to ensure the robustness of our conclusions. Third, our use of weighted statistical analysis better reflects the health status of the American population. Finally, categorizing individuals into four groups based on muscle and bone status minimized the potential interference between sarcopenia and low BMD.

This study also has several limitations. First, As the number of high-quality prospective cohort or randomized controlled studies on osteosarcopenia remains limited, robust evidence to determine causal relationships with specific variables is lacking. Therefore, our covariate selection was primarily based on existing literature and clinical reasoning. Nevertheless, it is possible that unmeasured or residual confounding remains. Second, the cross-sectional design limits our ability to determine a causal relationship between the OBS and osteosarcopenia. Third, the findings are based on an analysis of the American population, necessitating further validation in other regions.

## Conclusion

OBS is negatively associated with the prevalence of osteosarcopenia and may serve as a useful marker for identifying individuals at higher risk. Its potential role in early risk stratification warrants further exploration. Prospective cohort studies are warranted to further investigate the potential causal relationship between OBS and osteosarcopenia.

## Data Availability

Publicly available datasets were analyzed in this study. This data can be found here: https://www.cdc.gov/nchs/nhanes/.

## Footnotes

1
https://www.cdc.gov/nchs/nhanes/index.html
