# Supplementary material for: Association between oxidative balance score and osteosarcopenia in US adults: insights from a population-based study
Source: Front Nutr. 2025 Jul 31;12:1612406. doi: 10.3389/fnut.2025.1612406 (PMC12352320; doi:10.3389/fnut.2025.1612406)
Supplement: Supplementary file 1 [file Table_1.docx]

**Supplementary materials**

Association between oxidative balance score and osteosarcopenia in US adults: insights from a population-based study

**Table S1.** Scheme for the assignment of OBS.

| **OBS components** | **Property** | **Male** | | | **Female** | | |
| --- | --- | --- | --- | --- | --- | --- | --- |
|  |  | **0** | **1** | **2** | **0** | **1** | **2** |
| **Dietary OBS** | | | | | | | |
| Dietary fiber (g/d) | A | <12.10 | 12.10–20.50 | ≥20.50 | <9.80 | 9.80–16.20 | ≥16.20 |
| Carotene (RE/d) | A | <33. 81 | 33.81–108.00 | ≥108.00 | <33.26 | 33.26–132.30 | ≥132.30 |
| Riboflavin (mg/d) | A | <1.72 | 1.72–2.66 | ≥2.66 | <1.30 | 1.30–2.01 | ≥2.01 |
| Niacin (mg/d) | A | <21.82 | 21.82–33.78 | ≥33.78 | <15.06 | 15.06–23.13 | ≥23.13 |
| Total folate (mcg/d) | A | <318.00 | 318.00–499.10 | ≥499.10 | <236.00 | 236.00–378.00 | ≥378.00 |
| Calcium (mg/d) | A | <661.00 | 661.00–1123.10 | ≥1123.10 | <544.27 | 544.27–937.00 | ≥937.00 |
| Zinc (mg/d) | A | <9.43 | 9.43–14.96 | ≥14.96 | <6.67 | 6.67–10.65 | ≥10.65 |
| Magnesium (mg/d) | A | <253.00 | 253.00–375.00 | ≥375.00 | <195.00 | 195.00–288.00 | ≥288.00 |
| Copper (mg/d) | A | <1.07 | 1.07–1.59 | ≥1.59 | <0.84 | 0.84–1.25 | ≥1.25 |
| Selenium (mcg/d) | A | <98.80 | 98.80–150.81 | ≥150.81 | <69.60 | 69.60–106.40 | ≥106.40 |
| Iron (mg/d) | P | ≥19.16 | 12.51–19.16 | <12.51 | ≥14.33 | 9.16–14.33 | <9.16 |
| Total fat (g/d) | P | ≥108.70 | 69.97–108.70 | <69.97 | ≥79.48 | 51.71–79.48 | <51.71 |
| Vitamin B6 (mg/d) | A | <1.63 | 1.63–2.59 | ≥2.59 | <1.15 | 1.15–1.85 | ≥1.85 |
| Vitamin B12 (mcg/d) | A | <3.20 | 3.20–6.31 | ≥6.31 | <2.07 | 2.07–4.26 | ≥4.26 |
| Vitamin C (mg/d) | A | <31.00 | 31.00–99.11 | ≥99.11 | <28.03 | 28.03–84.01 | ≥84.01 |
| Vitamin E (ATE) (mg/d) | A | <5.57 | 5.57–9.43 | ≥9.43 | <4.45 | 4.45–7.53 | ≥7.53 |
| **Lifestyle OBS** | | | | | | | |
| Physical activity (MET-minute/week) | A | <400.00 | 400.00–1680.00 | ≥1680.00 | <409.50 | 409.50–1440.00 | ≥1440.00 |
| Body mass index (kg/m^2^) | P | ≥29.10 | 25.30–29.10 | <25.30 | ≥29.59 | 24.19–29.59 | <24.19 |
| Alcohol (g/d) | P | ≥30 | 0–30 | None | ≥15 | 0–15 | None |
| Cotinine (ng/mL) | P | ≥8.38 | 0.03–8.38 | <0.03 | ≥0.13 | 0.02–0.13 | <0.02 |

OBS: oxidative balance score; A: antioxidant; P: prooxidant; RE: retinol equivalent; ATE: alpha-tocopherol equivalent; MET: metabolic equivalent.

**Table S2.** The association of low bone mineral density, sarcopenia and osteosarcopenia with oxidative balance score levels.

|  | **Model 1** | |  | **Model 2** | |  | **Model 3** | |
| --- | --- | --- | --- | --- | --- | --- | --- | --- |
|  | **β (95%CI)** | **P-value** |  | **β (95%CI)** | **P-value** |  | **β (95%CI)** | **P-value** |
| **Osteosarcopenia** | -2.51(-4.11,-0.91) | **0.003** |  | -1.86(-3.54,-0.19) | **0.030** |  | -1.75(-3.43,-0.06) | **0.043** |
| **Sarcopenia** | -3.78(-5.18,-2.39) | **<0.001** |  | -2.95(-4.46,-1.44) | **<0.001** |  | -2.68(-4.13,-1.22) | **<0.001** |
| **Low bone mineral density** | -0.21(-0.92,0.51) | 0.565 |  | 0.00(-0.69, 0.68) | 0.990 |  | 0.02(-0.65, 0.69) | 0.948 |

Model 1: Unadjusted model.

Model 2: Adjusted for age group, gender, race, marital status, poverty-income ratio, and education level.

Model 3: Additionally, adjusted for hypertension, diabetes, and hyperlipidemia.
CI, confidence interval.

Table S3. Sensitivity analyses for the association between OBS and osteosarcopenia.

|  | **Model 4** | |  | **Model 5** | |  | **Model 6** | |  | **Model 7** | |
| --- | --- | --- | --- | --- | --- | --- | --- | --- | --- | --- | --- |
|  | **OR (95%CI)** | ***P*-value** |  | **OR (95%CI)** | ***P*-value** |  | **OR (95%CI)** | ***P*-value** |  | **OR (95%CI)** | ***P*-value** |
| **OBS** | 0.96(0.92, 1.00) | **0.029** |  | 0.96(0.93, 1.00) | **0.042** |  | 0.96(0.92, 1.00) | **0.031** |  | 0.96(0.93, 1.00) | **0.038** |
| **Q1** | ref |  |  | ref |  |  | ref |  |  | ref |  |
| **Q2** | 0.88(0.47, 1.67) | 0.691 |  | 0.89(0.48, 1.63) | 0.685 |  | 0.84(0.45, 1.56) | 0.558 |  | 0.88(0.48, 1.62) | 0.664 |
| **Q3** | 0.86(0.47, 1.56) | 0.607 |  | 0.89(0.52, 1.54) | 0.672 |  | 0.83(0.45, 1.52) | 0.525 |  | 0.88(0.50, 1.55) | 0.651 |
| **Q4** | 0.24(0.11, 0.54) | **0.001** |  | 0.27(0.12, 0.60) | **0.002** |  | 0.25(0.11, 0.56) | **0.001** |  | 0.26(0.11, 0.58) | **0.002** |
| ***P* for trend** |  | **0.003** |  |  | **0.005** |  |  | **0.004** |  |  | **0.005** |

Model 4: Additionally adjusted for dietary supplement use.

Model 5: Additionally adjusted for vitamin D status.

Model 6: Additionally adjusted for inflammatory markers.

Model 7: Additionally adjusted for dietary supplement use, vitamin D status, and inflammatory markers.

Bold indicates a P-value less than 0.05. OBS, oxidative balance score; OR, odds ratio; CI, confidence interval.

Notes: Dietary supplements were assessed using a structured questionnaire that asked participants, “Any dietary supplements taken?”, with response options of Yes or No. Vitamin D status was determined based on total serum 25-hydroxyvitamin D [25(OH)D] levels, measured using a high-performance liquid chromatography–tandem mass spectrometry (HPLC–MS/MS) technique. Participants were categorized as non-deficient if 25(OH)D ≥50 nmol/L, and as deficient otherwise. Inflammation status was adjusted using three novel composite inflammatory indices, which are considered reliable markers of systemic inflammation and immune activation: SIRI: systemic inflammation response index = (NEU × MONO) / LYM, SII: systemic immune-inflammation index = (NEU × PLT) / LYM, AISI: aggregate index of systemic inflammation = (NEU × MONO × PLT) / LYM.
